# Transcriptome Analysis of *Dendrobium officinale* and its Application to the Identification of Genes Associated with Polysaccharide Synthesis

**DOI:** 10.3389/fpls.2016.00005

**Published:** 2016-02-05

**Authors:** Jianxia Zhang, Chunmei He, Kunlin Wu, Jaime A. Teixeira da Silva, Songjun Zeng, Xinhua Zhang, Zhenming Yu, Haoqiang Xia, Jun Duan

**Affiliations:** ^1^Key Laboratory of South China Agricultural Plant Molecular Analysis and Gene Improvement, South China Botanical Garden, Chinese Academy of SciencesGuangzhou, China; ^2^Independent ResearcherKagawa-ken, Japan; ^3^Guangzhou Genedenovo Biotechnology Co., LtdGuangzhou, China

**Keywords:** *D. officinale*, glycosyltransferase, secondary metabolism, polysaccharide synthesis, transcriptome

## Abstract

*Dendrobium officinale* is one of the most important Chinese medicinal herbs. Polysaccharides are one of the main active ingredients of *D. officinale*. To identify the genes that maybe related to polysaccharides synthesis, two cDNA libraries were prepared from juvenile and adult *D. officinale*, and were named Dendrobium-1 and Dendrobium-2, respectively. Illumina sequencing for Dendrobium-1 generated 102 million high quality reads that were assembled into 93,881 unigenes with an average sequence length of 790 base pairs. The sequencing for Dendrobium-2 generated 86 million reads that were assembled into 114,098 unigenes with an average sequence length of 695 base pairs. Two transcriptome databases were integrated and assembled into a total of 145,791 unigenes. Among them, 17,281 unigenes were assigned to 126 KEGG pathways while 135 unigenes were involved in fructose and mannose metabolism. Gene Ontology analysis revealed that the majority of genes were associated with metabolic and cellular processes. Furthermore, 430 glycosyltransferase and 89 cellulose synthase genes were identified. Comparative analysis of both transcriptome databases revealed a total of 32,794 differential expression genes (DEGs), including 22,051 up-regulated and 10,743 down-regulated genes in Dendrobium-2 compared to Dendrobium-1. Furthermore, a total of 1142 and 7918 unigenes showed unique expression in Dendrobium-1 and Dendrobium-2, respectively. These DEGs were mainly correlated with metabolic pathways and the biosynthesis of secondary metabolites. In addition, 170 DEGs belonged to glycosyltransferase genes, 37 DEGs were related to cellulose synthase genes and 627 DEGs encoded transcription factors. This study substantially expands the transcriptome information for *D. officinale* and provides valuable clues for identifying candidate genes involved in polysaccharide biosynthesis and elucidating the mechanism of polysaccharide biosynthesis.

## Introduction

The *Orchidaceae* is one of the largest and most widespread families of flowering plants, with more than 250,000 species (Leitch et al., 2009). The genus *Dendrobium* is one of the largest genera of the *Orchidaceae* and has nearly 1100 species throughout the world and is spread widely in India across to Japan, south to Malaysia, and east to Australia, New Guinea, and the Pacific islands (Wu et al., 2009). *Dendrobium officinale*, a critically endangered orchid in the wild (http://www.iucnredlist.org/details/46665/0), has been one of the most important Chinese herbs in China for hundreds of years and is eaten or used as folk medicine for antipyretic, eye-benefitting and immune regulatory purposes (Yang et al., 2006).

The major active ingredients of *D. officinale* are polysaccharides, alkaloids, phenols, coumarins, terpenes, flavonoids, amino acids, benzyl compounds, and several trace mineral elements (Weng, 2003; Li et al., 2011). *D. officinale* has a thick water-soluble polysaccharide-rich stem. *Dendrobium* polysaccharides are mainly composed of glucose and mannose, as well as a small amount of rhamnose, xylose, and arabinose (Fan et al., 2009; Luo et al., 2010). Polysaccharides have been demonstrated in recent years to show prominent bioactivities, including antioxidant, immune stimulation, and anti-tumor (Hsieh et al., 2008; Fan et al., 2009; Luo et al., 2010; Wang et al., 2010; Liu et al., 2011; Xia et al., 2012). Soluble polysaccharides from *D*. *officinale* exerted stronger immune modulatory activity than *D*. *fimbriatum, D*. *nobile, D*. *chrysotoxum*, and *D. huoshanense* (Meng et al., 2013). *Dendrobium* polysaccharides have gained increasing attention in the biomedical and drug delivery fields. On the current market, the quality of *D. officinale* is mainly determined by the content of soluble polysaccharides. The component of polysaccharides from different *Dendrobium* species is different. For example, the polysaccharide fractions from *D. denneanum* are composed of glucose, mannose and galactose in the ratio of 227:59:17, as well as small amounts of xylose and arabinose (Fan et al., 2009). The polysaccharide fraction from *D. huoshanense* consists of glucose, mannose and galactose in the ratio of 31:10:8 (Zha et al., 2007). The polysaccharides from *D. officinale* were shown to be a 2-*O*-acetylglucomannan, composed of mannose, glucose, and arabinose in a 40.2:8.4:1 molar ratio (Hua et al., 2004). On the whole, mannose and glucose are the main monosaccharides in these *Dendrobium* species. Although the bioactivities, composition, structure, and physicochemical properties of polysaccharides from *Dendrobium* are well defined, the enzymes and encoding genes responsible for their synthesis and metabolic pathway remain poorly characterized. Therefore, an understanding of the molecular mechanisms underlying the synthesis of *Dendrobium* polysaccharides is essential.

So far, the transcriptome of only one *Dendrobium* species has been sequenced (Guo et al., 2013). It only revealed limited information related to genes in the stem in a certain stage, focusing on the putative alkaloid biosynthetic genes and genetic markers. The molecular mechanisms underlying polysaccharides synthesis and the related metabolic pathway for *D. officinale* remain unknown. In this study, we established two transcription databases for juvenile and adult *D. officinale* and identified 430 glycosyltransferase genes (GTs) and 89 cellulose synthase genes (CesA). Differentially expressed genes (DEGs) were analyzed. Differentially expressed GTs, CesA and transcription factors (TFs) are also reported. Such data for *D. officinale* could be used as an important resource to investigate GTs and the metabolic pathway of polysaccharides in *D*. *officinale*. Furthermore, this database will supply important clues to explore other biological mechanisms in this *Dendrobium* species and in other orchids.

## Materials and methods

### Plant materials and growth conditions

**Figure 1:**
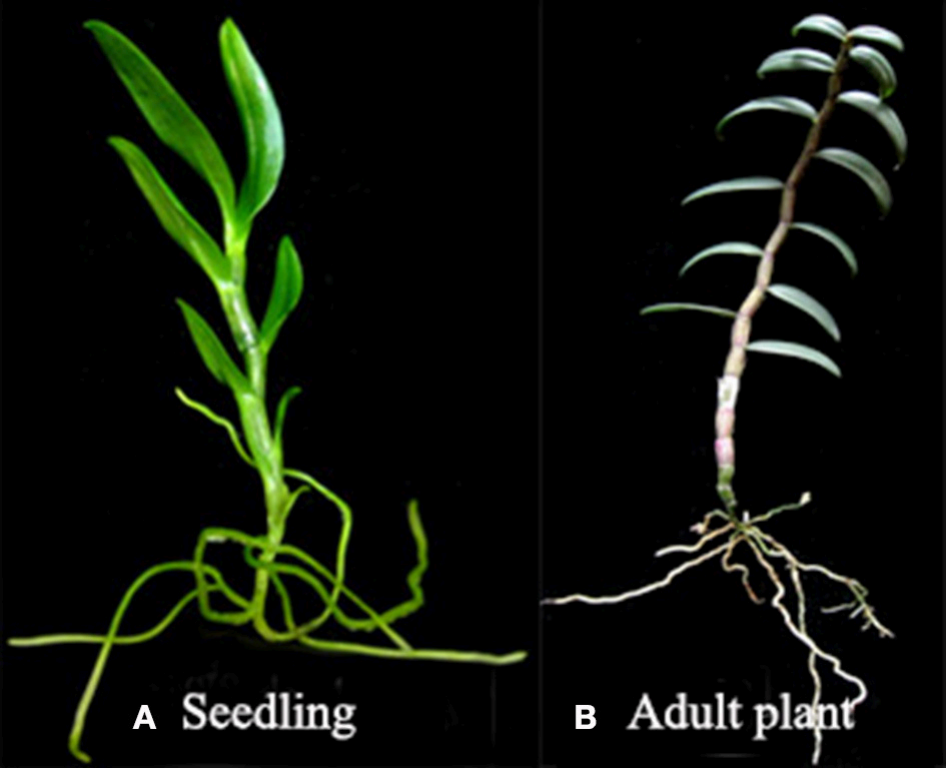
**Organs from *D. officinale* used to prepare cDNA libraries for Illumine sequencing**. **(A)** Young seedling *of D. officinale* (Dendrobium-1); **(B)** Adult plant of *D. officinale* (Dendrobium-2).

*D. officinale* was grown in the greenhouse of the South China Botanical Garden and used in this study. Seeds derived from selfing were germinated and cultured on half-strength Murashige and Skoog (MS) (Murashige and Skoog, 1962) medium containing 0.1% activated carbon, 2% sucrose, and 0.6% agar (pH = 5.4). The cultures were incubated at 26 ± 1°C with a 12 h photoperiod under cool white fluorescent lamps delivering a photosynthetic photon flux density (PPFD) of *ca*. 45 μmol m^−2^s^−1^. Material was collected from juvenile seedlings and adult plants. The juvenile seedlings were 10 months old after germinating *in vitro*. The adult plants were 18 months old after juvenile seedlings was transplanted into pots and placed in the greenhouse of the South China Botanical Garden at a day/night temperature of 28/25°C with a 12-h period. The materials used are shown in Figure 1. cDNA libraries were prepared from entire *D. officinale* plants at the juvenile and adult stages. Plants were collected in November 2013 at the vegetative stage.

Fresh samples were used to extract total RNA immediately.

### cDNA library preparation and illumina sequencing for transcriptome analysis

Total RNA (25 μg) was extracted using Column Plant RNAout 2.0 (Tiandz Inc., Beijing, China) according to the manufacturer's protocol. Preparation of the cDNA library was described in detail in a previous study employed for another orchid, *Cymbidium sinense* (Zhang et al., 2013). Two cDNA libraries were constructed from juvenile seedlings and adult plants in which equal amounts of total RNA were pooled from three biological replicates. The library of the juvenile seedlings was named Dendrobium-1 while the library of the adult plants was named Dendrobium-2. The two libraries were used for comparative analysis of transcriptome sequencing. Finally, two libraries were sequenced using the Illumina HiSeq™ 2000 platform at the Shenzhen Genome Institute (BGI, Shenzhen, China) and reads were generated in a 100 bp paired-end format according to the manufacturer's instructions (Illumina Inc. San Diego, CA). All raw transcriptome data were deposited in the GeneBank Short Read Archive. The accession numbers were SRR1904494 and SRR1909493 for Dendrobium-1 and Dendrobium-2, respectively.

### *De novo* assembly and functional annotation analysis of illumina sequencing

Raw reads from the sequencing machine were generated by base calling. After filtering raw reads by removing adaptor sequences, empty reads, reads with unknown nucleotides larger than 5% and low quality reads (with ambiguous sequences “N”), clean reads were obtained for *de novo* assembly. *De novo* assembly of the transcriptome was carried out with Trinity (*ver.* 2012-10-05) with the default parameters to form contigs (Grabherr et al., 2011). These contigs were then further processed with sequence clustering software, TGICL (Pertea et al., 2003), to form longer sequences defined as unigenes. The generated unigenes were used for BLASTX alignment (*E* < 0.00001) and annotation against protein databases, including non-redundant (nr), Swiss-Port, COG, and KEGG protein databases. With nr annotation, the Blast2GO program (Conesa et al., 2005) was used to obtain the Gene ontology (GO) annotation of unigenes, then WEGO software (Ye et al., 2006) was used to perform GO functional classification for all unigenes and to understand the distribution of gene functions. KEGG is a major public pathway-related database (Kanehisa et al., 2008) that is able to analyze a gene product during a metabolic process and related gene function in cellular processes. KEGG pathway annotation was performed using a BLAST search against the KEGG database (KEGG, http://www.genome.jp/kegg/).

### Identification of differentially expressed genes (DEGs)

To compare the differences in gene expression at two developmental stages, the RPKM method (reads per kb per million reads) was used to calculate read density. By taking into account the variations in gene length and the total mapped number of sequencing reads, the RPKM measure provides normalized values of gene expression that enable transcript comparisons between samples. The false discovery rate (FDR) was used to determine the threshold *P*-value in multiple tests. We used an FDR < 0.001, *P* ≤ 0.05 and an absolute value of the log_2_ ratio >1 as the threshold to determine significant differences in gene expression. The DEGs were used for GO and KEGG enrichment analyses according to a method used for *Cymbidium sinense* (Zhang et al., 2013).

### Quantitative real-time PCR validation

Total RNA was extracted as indicated above. Each RNA sample was treated with RNase-free DNase (Promega, Madison, USA) following the manufacturer's protocol in an effort to remove any residual genomic DNA (gDNA). DNase-treated RNA (2 mg) was subjected to reverse transcriptase reactions using M-MLV reverse transcriptase (Promega, Madison, USA) according to the manufacturer's instructions. The sequences of the specific primer sets are listed in Additional file 1. The constitutively expressed gene, *D*. *officinale actin* (cloned by our laboratory; NCBI accession number: JX294908), was used as the internal control. qRT-PCR was performed according to our previously published study (He et al., 2015). The expression level was calculated as 2^−ΔΔCt^ and normalized to the Ct value of *D*. *officinale actin*. The qRT-PCR results were obtained from three biological replicates and three technical repeats for each gene and sample.

## Results

### *De novo* assembly and sequence annotation

**Figure 2:**
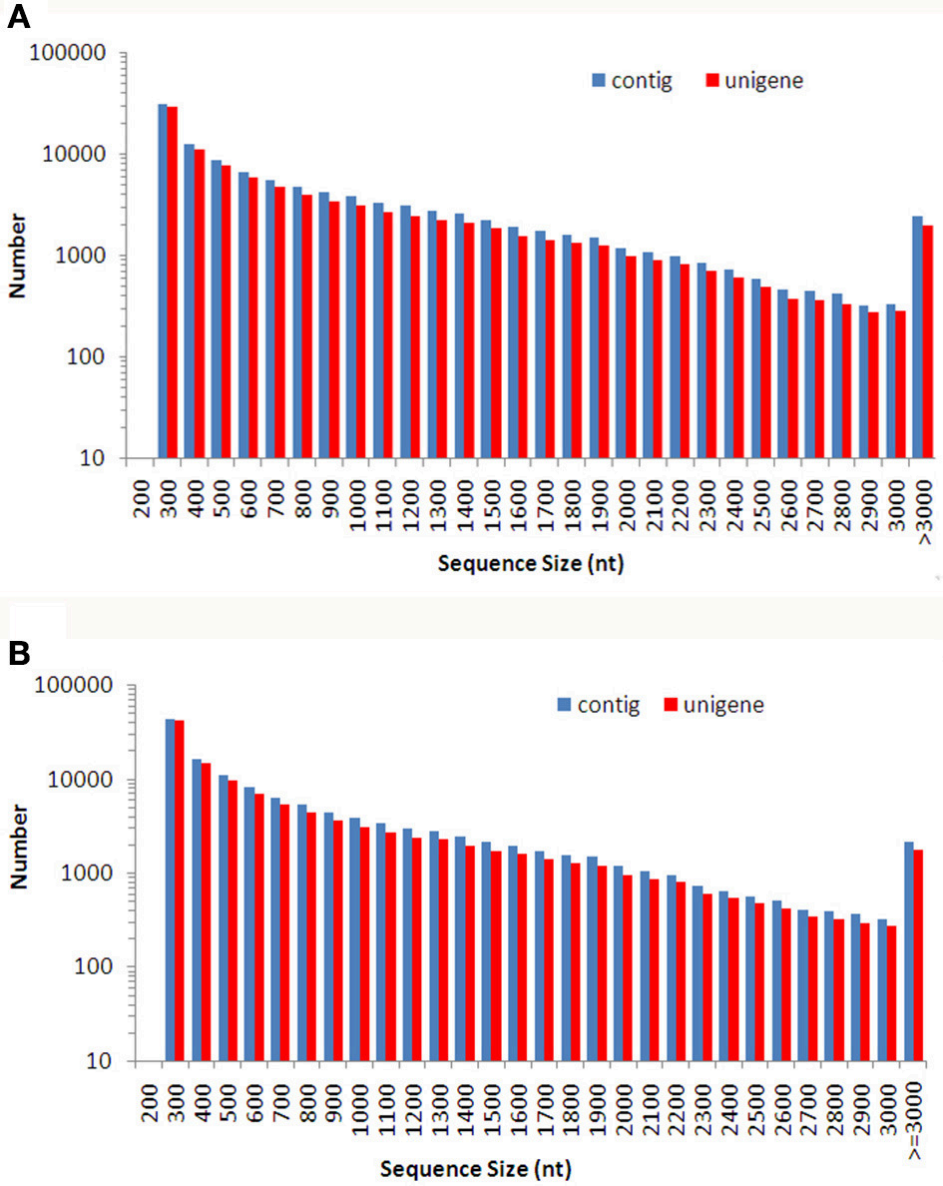
**The size distribution of *de novo* assembled contigs and unigenes for Dendrobium-1 (A) and Dendrobium-2 (B)**. A total of 107,086 contigs and 93,881 unigenes sizes were calculated for Dendrobium-1 **(A)**. A total of 129,235 contigs and 114,098 unigenes sizes were calculated for Dendrobium-2 **(B)**.

A total of 102 million 100 bp reads were assembled into 107,086 contigs with a mean length of 824 bp in Dendrobium-1, and a total of 86 million 100 bp reads were assembled into 129,235 contigs with a mean length of 728 bp in Dendrobium-2 (Table 1). Using paired-end reads, the Dendrobium-1 contigs were further assembled into 93,881 unigenes by Trinity with a mean length of 790 bp. The size distribution of these contigs and unigenes in Dendrobium-1 are shown in Figure 2A. The assembly produced a substantial number of large contigs and unigenes: 34,113 contigs were >1000 bp in length and 27,968 unigenes were >1000 bp in length (Figure 2A). The contigs in Dendrobium-2 were further assembled into 114,098 unigenes by Trinity with a mean length of 695 bp. The size distribution of these contigs and unigenes are shown in Figure 2B. The assembly produced a substantial number of large contigs and unigenes: 33,624 contigs were >1000 bp in length and 27,229 unigenes were >1000 bp in length (Figure 2B).

**Table 1:** **Summary of Illumina sequencing and assembly of two *D. officinale* transcriptomes**.

	**Dendrobium-1**	**Dendrobium-2**
Total number of raw reads	114,970,718	102,340,806
Total number of clean reads	102,982,138	86,515,904
Total clean nucleotides (nt)	10,298,213,800	8,651,590,400
Average read length	100	100
Total number of contigs	107086	129235
Mean length of contigs	824	728
Total number of unigenes	93881	114098
Mean length of unigenes	790	695

The contigs in two transcriptome sequencing databases were integrated and assembled into a total of 145,791 unigenes. These unigenes were annotated using BLASTX searches against NCBI, Nr, Swiss-Prot, KEGG, and COG databases. In total, there were 67,396 annotated unigenes (46.23% of all unigenes), providing a significant BLAST result. Among them, 66,541 unigenes (98.73% of all annotated unigenes) showed significant similarity to known proteins in the Nr database and 25,982 unigenes (38.55%) were annotated in COG based on sequence homologies.

**Figure 3:**
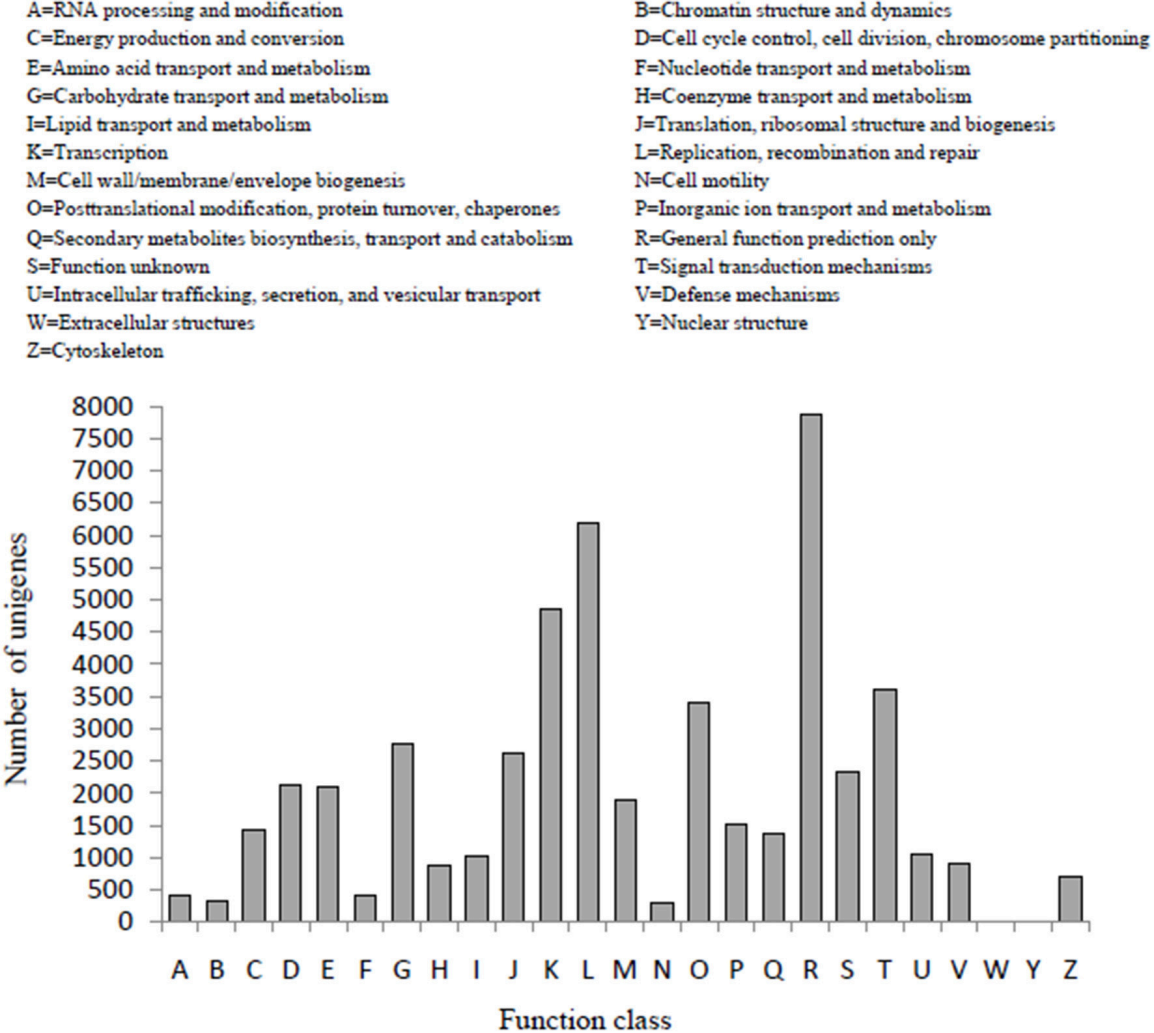
**COG function classification of *D. officinale* unigenes**. A total of 25,982 unigenes were classified into 25 functional categories according to their predicted gene products using the COG database (cut-off *E*-value of 0.00001).

In the COG classification, 25,982 unigenes were classified into 25 functional classifications (Figure 3). The most dominant term was “General function prediction only” and 7861 unigenes (30%) matched it. “Translation,” “replication, recombination, and repair” also shared a high percentage of genes among the categories, and only 4 and 19 unigenes matched the terms “nuclear structure” and “extracellular structures,” respectively. In addition, 2754 unigenes were annotated as the “carbohydrate transport and metabolism” category and 1386 unigenes in the “secondary metabolites biosynthesis transport and catabolism” category, both of which may play an important role in the biosynthesis of polysaccharides and small molecules with proven bioactivity.

### Gene ontology classification and metabolic pathway assignment by KEGG

**Figure 4:**
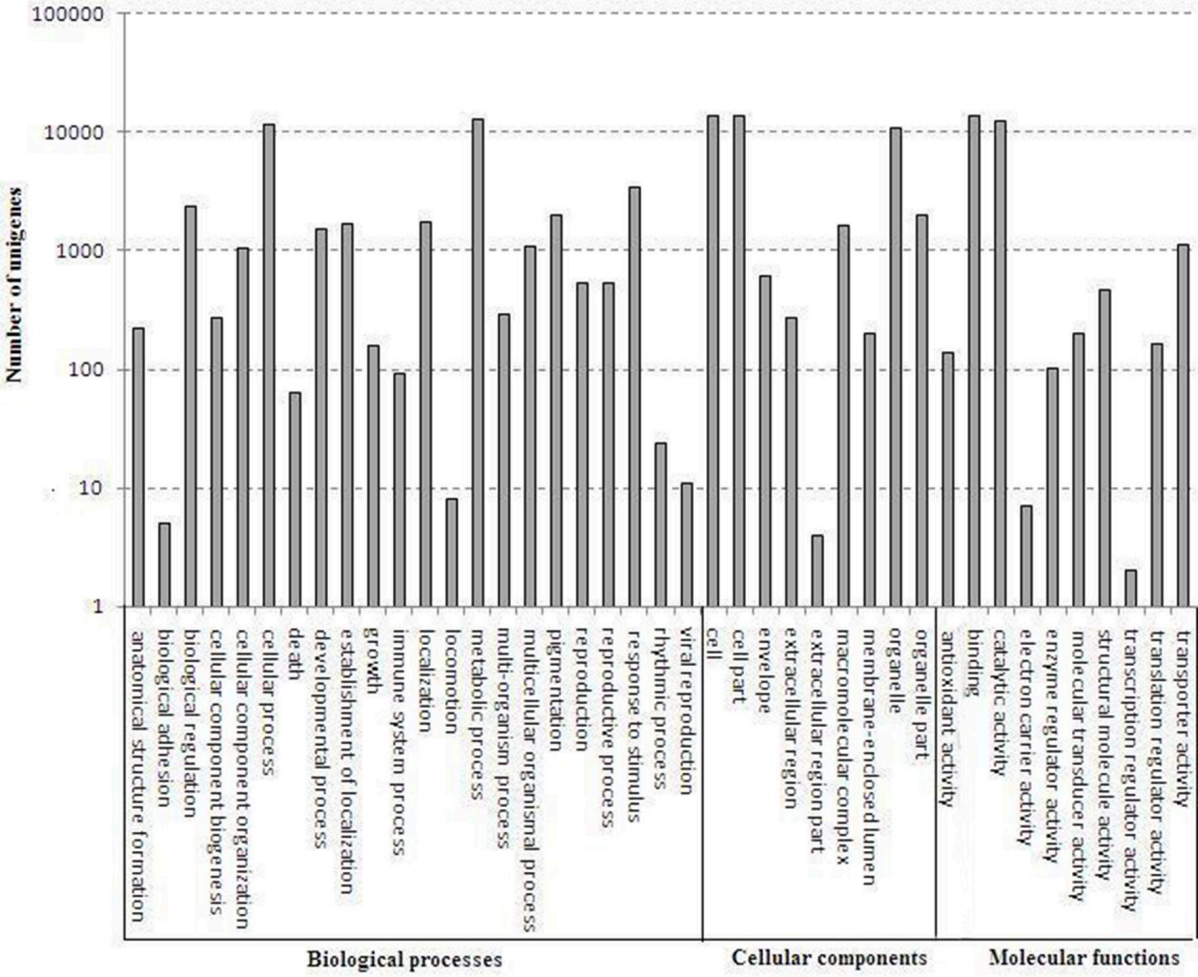
**Gene ontology categories of *D. officinale* unigenes**. The results are summarized in mainly three categories: biological process, cellular component and molecular function.

A total of 24,002 annotated unigenes were grouped into 41 functional groups by using GO assignments. Among these groups, 22 groups were involved in biological processes, 9 groups in cellular components and 10 groups in molecular functions. Metabolic processes and cellular processes were dominant in the biological process category. Within the molecular function category, a high percentage of genes were associated with catalytic activity and binding. Most assignments in cellular components were to cell components and cell membranes (Figure 4).

In this study, a total of 67,396 annotated sequences were mapped to reference canonical pathways in KEGG. In total, 17,281 sequences were assigned to 126 KEGG pathways (Additional file 2). The metabolic pathways represented the greatest group (4473 unigenes, or 25.88%), with most unigenes involved in starch and sucrose metabolism (320 unigenes), amino sugar and nucleotide sugar metabolism (288 unigenes), fructose and mannose metabolism (135 unigenes), and galactose metabolism (124 unigenes). A total of 2115 unigenes were involved in the biosynthesis of secondary metabolites, including phenylpropanoid biosynthesis, terpenoid backbone biosynthesis, cyanoamino acid metabolism, carotenoid biosynthesis, and others (Table 2). These pathways provide a valuable resource for investigating specific processes, functions and pathways during *D. officinale* development.

**Table 2:** **The pathways and number of unigenes related to secondary metabolites in *D. officinale***.

**Biosynthesis of secondary metabolites pathway**	**All genes with pathway annotation (17281)**	**Pathway ID**
Anthocyanin biosynthesis	1 (0.01%)	ko00942
Benzoxazinoid biosynthesis	1 (0.01%)	ko00402
Betalain biosynthesis	2 (0.01%)	ko00965
Brassinosteroid biosynthesis	19 (0.11%)	ko00905
Caffeine metabolism	6 (0.03%)	ko00232
Carotenoid biosynthesis	70 (0.41%)	ko00906
Cyanoamino acid metabolism	87 (0.5%)	ko00460
Diterpenoid biosynthesis	52 (0.3%)	ko00904
Flavone and flavonol biosynthesis	20 (0.12%)	ko00944
Flavonoid biosynthesis	57 (0.33%)	ko00941
Indole alkaloid biosynthesis	4 (0.02%)	ko00901
Isoquinoline alkaloid biosynthesis	34 (0.2%)	ko00950
Monoterpenoid biosynthesis	4 (0.02%)	ko00902
Nicotinate and nicotinamide metabolism	27 (0.16%)	ko00760
Phenylpropanoid biosynthesis	216 (1.25%)	ko00940
Sesquiterpenoid biosynthesis	13 (0.08%)	ko00909
Steroid biosynthesis	49 (0.28%)	ko00100
Stilbenoid, diarylheptanoid, and gingerol biosynthesis	48 (0.28%)	ko00945
Terpenoid backbone biosynthesis	104 (0.6%)	ko00900
Tropane, piperidine, and pyridine alkaloid biosynthesis	38 (0.22%)	ko00960

**Figure 5:**
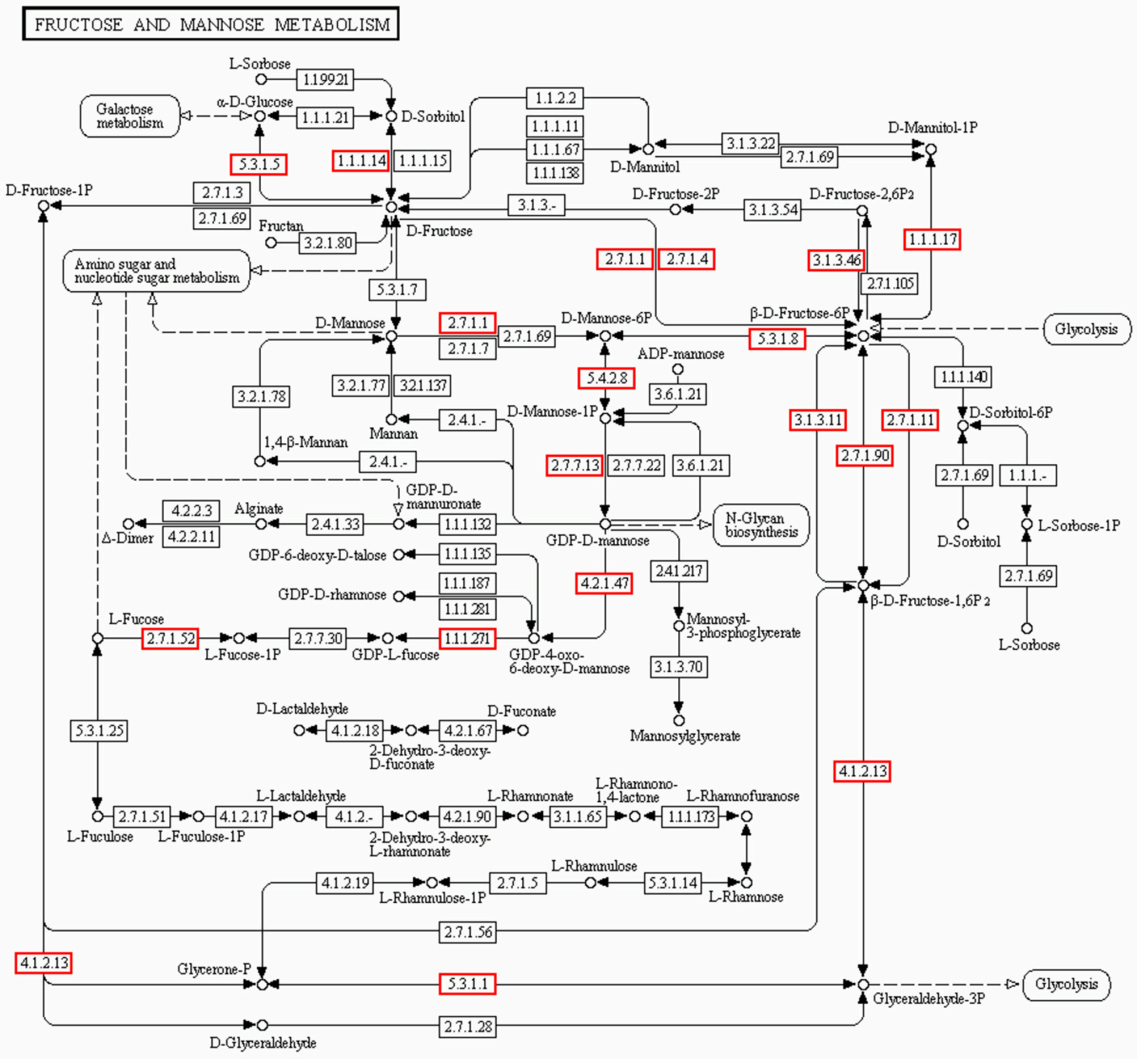
**Putative fructose and mannose metabolic pathway of *D.officinale.*** Putative fructose and mannose metabolism of *D.officinale* was constructed based on KEGG annotation. A total of 135 unigenes were involved in the metabolic pathway. These unigenes were distributed in the rectangular boxes in the figure.

Mannose and glucose are the main monosaccharide building blocks in *D. officinale.* Fructose and mannose metabolism found in the KEGG pathway involved 135 unigenes. A detailed metabolic pathway for fructose and mannose metabolism is shown in Figure 5. Every gene in the pathway was associated with several unigenes. The pathway will be useful for further studies on the effect of the fructose and mannose metabolism pathway on the biosynthesis of active polysaccharides.

### Identifying *D. officinale* glycosyltransferase genes and cellulose synthase genes

**Figure 6:**
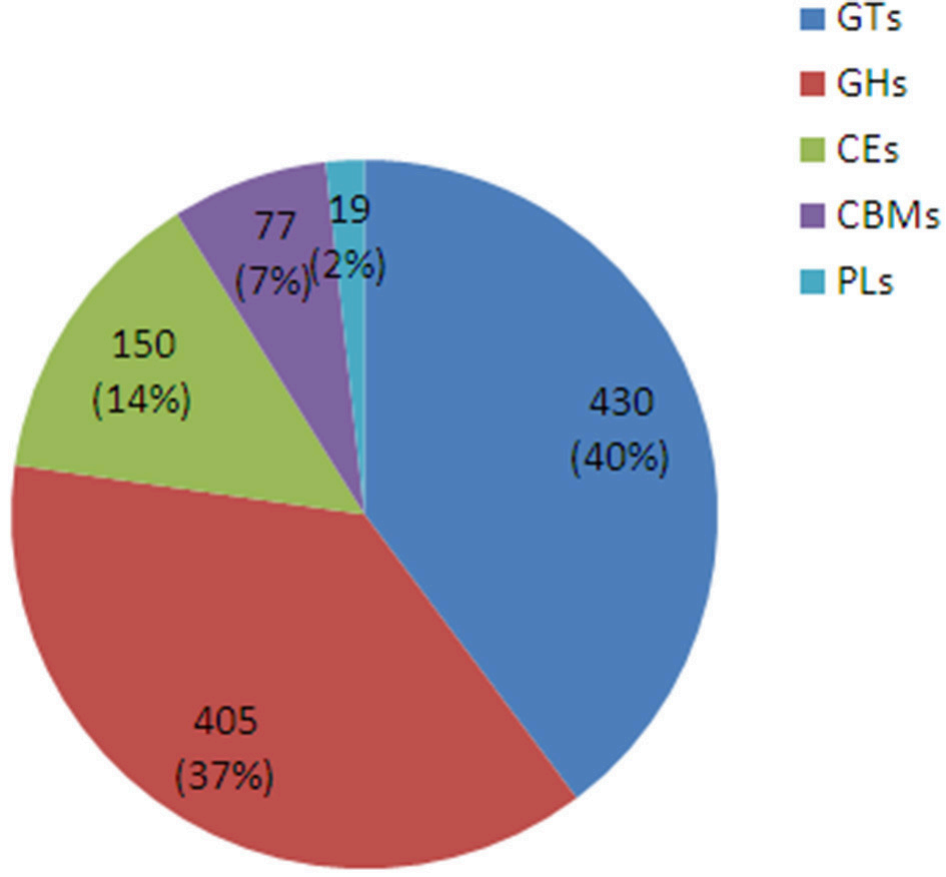
**The classification and number of carbohydrate-active enzyme families in *D. officinale* unigenes**. GT, Glycosyltransferase; GH, Glycoside Hydrolase; CE, Carbohydrate Esterase; CBM, Carbohydrate-Binding Module; PL, Polysaccharide Lyase.

A sequencing similarity search was conducted against the CAZy database by using BLASTX (*E* < 0.00001), identifying a total of 1081 carbohydrate-active related unigenes (Additional file 3), including 430 glycosyltransferase genes (GTs), 405 glycoside hydrolases, 150 carbohydrate esterases, 77 carbohydrate-binding modules, and 19 polysaccharide lyases (Figure 6).

GTs, which are enzymes that synthesize oligosaccharides, polysaccharides, and glycoconjugates, were dominant in carbohydrate-active related unigenes, and 430 GTs were divided into 35 GT families. A comparison of GTs families and numbers among *A. thaliana, O. sativa*, and *D. officinale* is shown in Additional file 4. *D. officinale* lacks several GT families, GT9, GT16, GT19, GT30, GT33, GT37, GT50, GT57, and GT58, which were present in *A. thaliana* and *O. sativa.* GT59 and GT76 were present in *A. thaliana* and *O. sativa*, but not in *D. officinale* while GT39 was present only in *D. officinale*.

**Figure 7:**
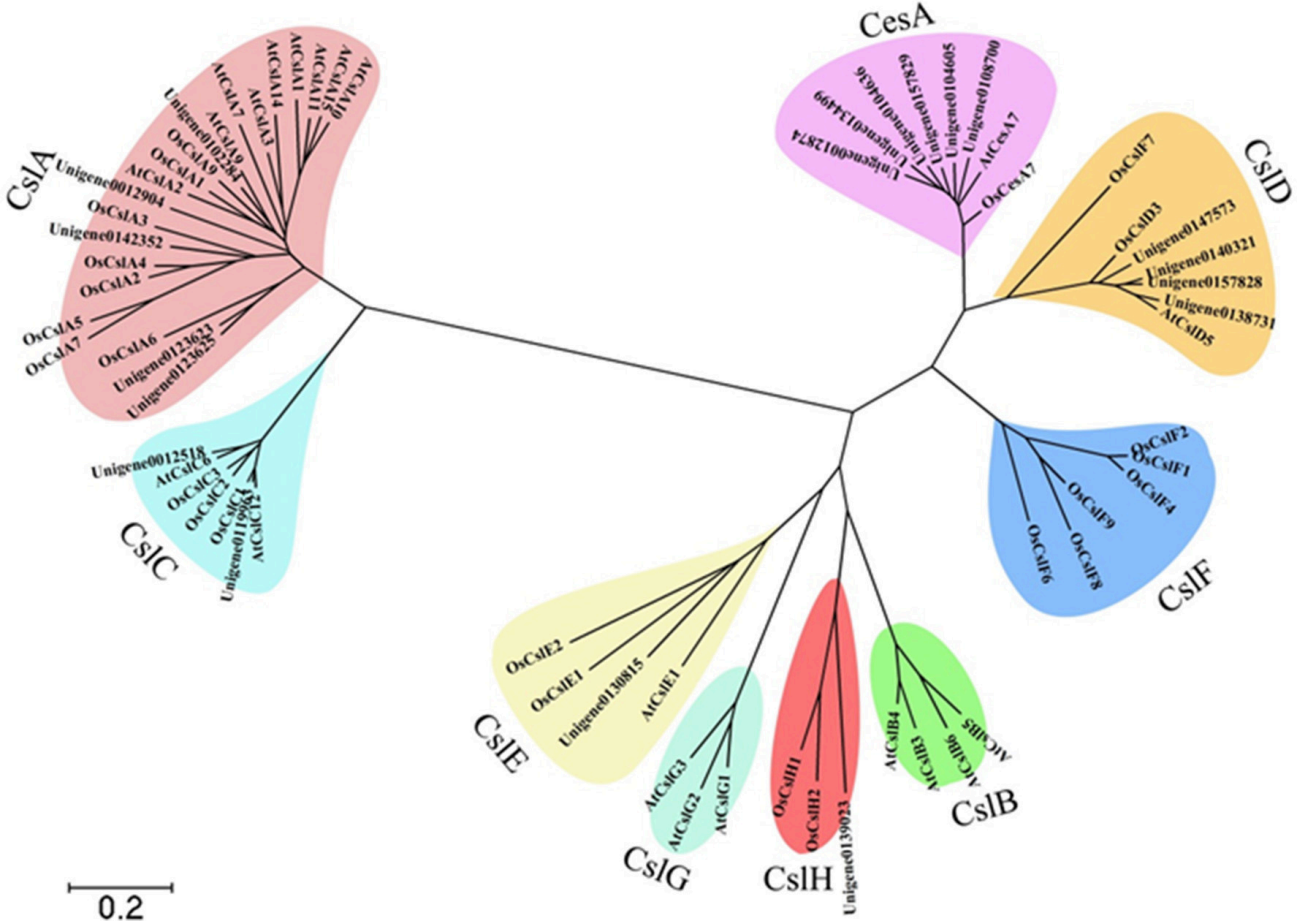
**Molecular phylogenetic tree of the amino acid sequences of the CesA superfamily of *D. officinale*, *A. thaliana*, and *O. sativa***. The tree was constructed using MEGA 4 by the neighbor-joining method. The tree was displayed as a phylogram in which branch lengths are proportional to distance. Bootstrap values for 1000 replicates were used to assess the robustness of the trees. The amino acid sequences of *A. thaliana* and *O. sativa* used for alignment are as follows: AtCesA7, gb|AAD32031.1|; AtCslA1, gb|AAO42230.1|; AtCslC12, gb|AAD15482.1|; AtCslG2, gbAAB63623.1|; AtCslA7, gb|AAL24081.1|; AtCslG3, gb|AAB63624.1|; AtCslG1, gb|AAB63622.1|; AtCslA15, gb|AEE83276.1; AtCslC6, gb|AAF02144.1|; AtCslD5, gb|AAF02892.1|; AtCslA3, gb|AAF79586.1|; AtCslB6, gb|AEE83584.1|; AtCslB5, gb|AAQ22621.1|; AtCslA9, gb|AAL31192.1|; AtCslA14, gb|AAO42815.1|; AtCslB4, gb|AAC25936.1|; AtCslB3, gb|AAC25935.1|; AtCslE1, gb|AAF79313.1|; AtCslA10, gb|AAF87149.1|; AtCslA2, gb|AAL24334.1|; AtCslA11, gb|AED92259.1|; OsCslA6, gb|AAL25127.1|; OsCslF1, gb|AAL25131.1|; OsCslE2, gb|AAL25130.1|; OsCslA9, gb|AAL25128.1|; OsCslA3, tpg|DAA01744.1|; OsCslD3, tpg|DAA01756.1|; OsCslC2, tpg|DAA01750.1|; OsCslF7, gb|AAK91320.1|; OsCslA2, gb|AAK98678.1|; OsCslF2, gb|AAL25132.1|; OsCslA5, gb|AAL82530.1|; OsCslA4, gb|AAL84294.1; OsCslH2, dbj|BAF14725.2|; OsCslC1, dbj|BAC10759.1|; OsCslH1, gb|AAN01252.1|; OsCslA7, gb|ABG34297.1|; OsCslF8, dbj|BAC65371.1|; OsCslF9, dbj|BAC80027.1|; OsCslF6, dbj|BAC66734.1|; OsCslD3, dbj|BAD01697.1|; OsCslF4, dbj|BAC83321.1|; OsCslC3, dbj|BAC98512.1|; OsCslA1, tpg|DAA01743.1|; OsCslE1, dbj|BAD46389.1|; OsCesA7, gb|AAK27814.1|.

The family of mannans is the most widespread group of polysaccharides in higher plants (Moreira and Filho, 2008). Cellulose synthase (CesA) superfamily genes were involved in the biosynthesis of mannan polysaccharides (Liepman et al., 2005). The CesA superfamily is classified into one CesA family and nine cellulose synthase-like (Csl) families, namely CslA/B/C/D/E/F/G/H/J. The CesA superfamily members of *A. thaliana* and *O. sativa* were used as bait for blasting the candidate unigenes from *D. officinale* protein libraries. A total of 89 candidate unigenes for CesA in *D. officinale* were identified and were listed in Additional file 5. A molecular phylogenetic tree (Figure 7) was constructed by using MEGA4 (Tamura et al., 2007), employing 19 unigenes that were translated into amino acid sequences, together with other CesA superfamily members from *A. thaliana* and *O. sativa*. The 19 unigenes were classified into six families, CesA, CslA, CslC, CslD, CslE, and CslH with 6 and 5 unigenes belonging to CesA and CslA families, respectively.

### Screening and identification of DEGs

To identify the DEGs during both developmental stages, the number of clean tags for each gene was calculated, and the genes that were differentially expressed between the two samples were identified according to the method described by Audic and Claverie (1997).

**Figure 8:**
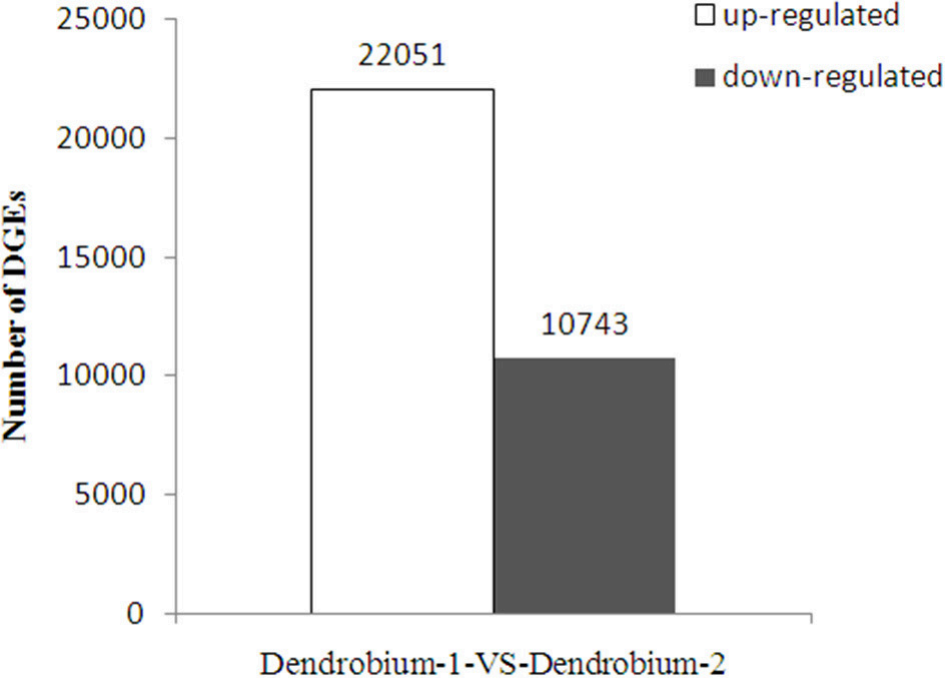
**Analysis of differentially expressed genes (DEGs) at two development stage**. The y-axis indicates the number of DEGs with an absolute value of the log_2_ ratio >1 between Dendrobium-1 vs. Dendrobium-2. The number of up-regulated and down-regulated genes between Dendrobium-1 vs. Dendrobium-2 are summarized.

**Figure 9:**
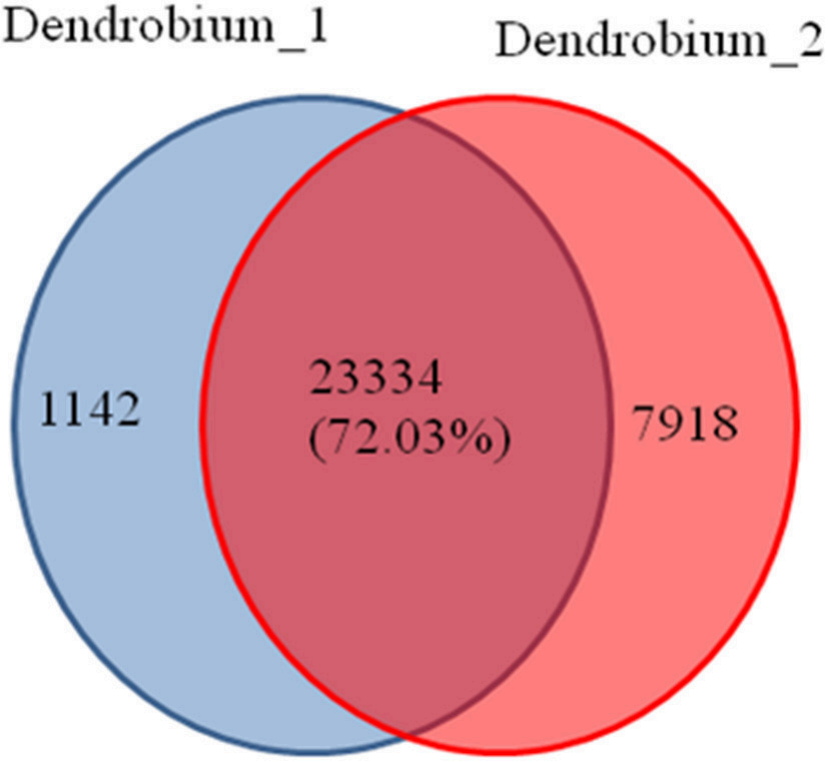
**Venn diagram of the unigenes at two development stage**. The diagram shows the overlapping unigenes at the two development stage. A total of 23334 (72.03%) unigenes were expressed in the Dendrobium-1 and Dendrobium-2. While 1142 unigenes were expressed only in the Dendrobium-1 and 7918 unigenes were expressed only in the Dendrobium-2.

A total of 32,794 DEGs were obtained, including 22,051 up-regulated and 10,743 down-regulated genes in Dendrobium-2 compared to Dendrobium-1 (Figure 8). Furthermore, 1142 and 7918 unigenes expressed uniquely in Dendrobium-1 and Dendrobium-2, respectively, and 23,334 unigenes were expressed in both libraries, but at different levels (Figure 9). These specific DEGs in Dendrobium-1 and Dendrobium-2 are shown in Additional files 6, 7, respectively.

Among 32,794 DEGs, a total of 18,517 (56.46% of all DEGs) unigenes provided a significant BLAST result. Approximately 4722 unigenes could be annotated in KEGG and 6356 unigenes could be annotated in GO based on sequence homologies, while 18046, 13790, and 7490 unigenes were annotated in Nr, SWISSprot, and COG, respectively.

In the KEGG classification, 4722 DEGs were significantly enriched in 42 pathways (Additional file 8). Most genes were correlated to metabolic pathways and biosynthesis of secondary metabolites. Gluconeogenesis was also significantly enriched. Furthermore, the number of up-regulated genes was more than the number of down-regulated genes in the three pathways.

The analysis of biological processes on these DEGs was performed based on GO functional classification. These genes, including specific unigenes in Dendrobium-1 and Dendrobium-2, as well as up-regulated and down-regulated unigenes in Dendrobium-1 vs. Dendrobium-2, were mainly correlated to metabolic and cellular processes and to responses to stimuli (Additional file 9). The main biological process for these DEGs was similar.

### DEGs related to glycosyltransferases genes, cellulose synthase genes, and transcription factors

Among 1081 carbohydrate-related genes, only 235 DEGs were identified, including 94 up-regulated and 141 down-regulated genes. These carbohydrate-related DEGs included 170 GT, 28 mannnosyltransferase, 23 galactosylatransferase, 10 xylosyltransferase, and 4 fucosyltransferase DEGs (Table 3). 170 DEGs related to GT belonged to 28 GT families (list was shown in Additional file 10). Among them, GT1 and GT2 were the main families, including 38 and 33 DEGs, respectively. In the GT1 family, 10 DEGs were up-regulated and 28 DEGs were down-regulated. In contrast, in the GT2 family, 17 and 16 DEGs were up- and down-regulated, respectively (Table 4). Among 89 CesA-related genes, 37 genes showed differential expression, including seven up-regulated and 30 down-regulated genes. These were classified into five CslA families, CslA, CslD, CslE, CslF, and CslG, respectively (Table 5).

**Table 3:** **The category and number of differentially expressed carbohydrate-related genes in DEGs database**.

**Category**	**Number of DEGs**	**Up-regulated**	**Down-regulated**
Glycosyltransferase	170	70	100
Mannosyltransferase	28	15	13
Galactosylatransferase	23	8	15
Xylosyltransferase	10	1	9
Fucosyltransferase	4	1	3
Summary	235	94	141

**Table 4:** **The category and number of GT families in the DEGs database**.

**Family**	**Number of DEGs**	**Up-regulated**	**Down-regulated**
GT1	38	10	28
GT2	33	17	16
GT4	8	2	6
GT8	12	5	7
GT10	1	1	0
GT14	4	1	3
GT17	2	1	1
GT20	1	1	0
GT21	2	0	2
GT22	1	1	0
GT23	4	0	4
GT28	3	3	0
GT29	2	2	0
GT31	8	1	7
GT32	2	2	0
GT34	1	0	1
GT35	1	0	1
GT39	1	1	0
GT41	19	8	11
GT43	2	1	1
GT47	1	0	1
GT48	5	5	0
GT51	2	0	2
GT61	4	1	3
GT66	1	1	0
GT68	1	0	1
GT77	5	4	1
GT92	6	2	4
Summary	170	70	100

**Table 5:** **The category and number of Csl family in the DEGs database**.

**Family**	**Number of DEGs**	**Up-regulated**	**Down-regulated**
CslA	13	0	13
CslD	9	0	9
CslE	4	4	0
CslF	8	0	8
CslG	3	3	0
Summary	37	7	30

TFs have been implicated in a variety of developmental and physiological roles in plants. More TFs have also been isolated and characterized for several plant secondary metabolic pathways. In our *D. officinale* DEGs database, a total of 627 putative transcripts encoding TFs were identified, including 301 up-regulated unigenes and 326 down-regulated unigenes (Table 6). They belonged to known TF families, the most abundant being the MYB family, including 82 unigenes. In addition, 75 DEGs belonged to the bHLH family, 66 to the AP2/ERF family, 60 to the WRKY family, 33 to the Homeobox famlily, 30 to the MADS family, 24 to the NAC family, and 23 to the bZIP family. All these TFs have been identified as positive or negative regulators in the biosynthesis of secondary metabolites in other plants (Grotewold et al., 1998; van der Fits and Memelink, 2000).

**Table 6:** **The type and number of transcription factor families identified in the DEGs database of *D. officinale***.

**Transcription factor factor**	**Number of unigenes**	**Up-regulated**	**Down-regulated**
MYB	82	35	47
bHLH	75	37	38
AP2/ERF	66	21	45
WRKY	60	32	28
Homeobox	33	15	18
MADS	30	18	12
NAC	24	14	10
bZIP	23	13	10
GATA	15	4	11
NFY	11	7	4
LHW	7	3	4
TCP	6	1	5
IIIA	5	4	1
HEC4	5	1	4
ROC	5	1	4
MED	4	1	3
GRAS	4	1	3
PACC	4	1	3
CIGR2	3	2	1
MBF1	3	2	1
VIP-like	3	0	3
trihelix	3	1	2
RF2b-like	3	1	2
MYC	3	1	2
ABI3	3	1	2
ATF1	2	2	0
HY5-like	2	0	2
other	144	96	81
Total number	627	301	326

### Validation and expression analysis of key enzyme genes

To validate changes in gene expression patterns, 18 key enzyme-encoding genes associated with GT biosynthesis, including GT1 (unigene0020469, unigene0022886, unigene0017200), GT2 (unigene0102284, unigene0157828, unigene0038137), GT8 (unigene0038402), GT28 (unigene0109540, unigene0134761), GT31 (unigene0127099), GT35 (unigene0104576), GT47 (unigene0112298), GT61 (unigene0150405, unigene0150401), and GT92 (unigene0157887), were randomly selected to examine gene expression using RT-qPCR. The two libraries exhibited differential expression and were identical to those obtained by sequencing (Additional file 11). Thus, the data generated in this study is sufficient to be used as a tool to investigate some genes related to polysaccharide synthesis and metabolism in *D. officinale*.

## Discussion

### Illumina sequencing and sequence annotation

*D. officinale* is a very important traditional Chinese herb within the *Orchidaceae*. Even though polysaccharides are one of the most important active constituents of *D. officinale*, little is known about the mechanisms responsible for polysaccharide synthesis and metabolism. The aims of this study were to generate a large amount of cDNA sequence data that would facilitate more detailed studies in *D. officinale*, and to identify the genes related to polysaccharide synthesis and metabolism. The availability of transcriptome data for *D. officinale* will meet the initial information needs for functional studies of this species and its relatives. In this study, two RNA-seq was performed using Illumina sequencing, which generated a total of 145,791 unigenes. A total of 67,396 (46.23%) unigenes provided a significant BLAST result. This information far exceeded that reported previously (Guo et al., 2013) and provides more adequate resources to study this *Dendrobium* species.

### Glycosyltransferase genes and their differential expression patterns in *D. officinale*

*D. officinale* has a thick and soluble polysaccharide-rich stem. The biosynthesis of polysaccharides involves the action of hundreds of different GTs, which catalyze the transfer of sugar moieties from activated donor molecules to specific acceptor molecules to form glycosidic bonds. At the same time, the glycosylation reactions have a cascading effect, which affect many aspects of plant growth and development. The most recent update of CAZy (http://www.cazy.org/GlycosylTransferases.html) indicates that GTs from diverse species can be classified into 97 families. A total of 463 and 571 GT genes had been listed in *A. thaliana* and *O. sativa*, assigned to 42 and 43 families, respectively. We identified 430 possible GTs in the *D. officinale* transcriptome database that were divided into 35 GT families (Additional file 4).

The category and proportion of GT genes are different in different plants. The function of each GT family also shows differences. GT1 is a major GT family in plants and is commonly known as UDP glycosyltransferase (UGT) (Weis et al., 2008). GT1 plays an indispensable role in the biosynthesis and modification of plant natural products (Jones and Vogt, 2001). In *A. thaliana* and *O. sativa*, GT1 is the major family (26 and 35%, respectively. The second group consists of GT2, GT8, GT31, and GT47 families, each accounting for approximately 6–9% of the genes. In *D. officinale*, the major GT families (GT1, GT2, and GT41) represent approximately 15–16% each of total GT genes. The third group consists of GT4 and GT8 (5 and 8%, respectively). The amount of GT41 in *D. officinale* exceeds that in *A. thaliana* and *O. sativa.* Moreover, GT51 and GT39 are specific to *D. officinale.* These specificities may reflect unique metabolic aspects of *D. officinale*.

In a previous study on *D. officinale*, polysaccharides were shown to be distributed in all organs, but mainly accumulated in stems while the soluble polysaccharide content changed in different developmental stages (He et al., 2015). The polysaccharide content in the stems of adult plants was higher than in seedlings but the content in the leaves and roots of adult plants was lower than in seedlings (He et al., 2015). In our present study, a total of 170 GTs showed differential expression in a comparison between adult plants and juvenile seedlings, including 70 up-regulated GTs and 100 down-regulated GTs (Table 3). The up-regulated GTs likely mainly accounted for the synthesis of soluble polysaccharides in adult plants while down-regulated GTs were probably used to build plant cell walls and other morphological structures in seedlings in the juvenile stage.

GT1 was the major gene among the down-regulated genes, and GT2 was the major gene among the up-regulated genes. The up-regulated and down-regulated GTs families contained 20 and 19 GT families, respectively. Furthermore, all DEGs, including up-regulated genes, down-regulated genes, as well as specific genes in adult plants or juvenile seedlings, showed similar metabolic pathways and biosynthesis of secondary metabolites (Additional file 9). All these results suggest that a variety of GTs together mediated the synthesis of soluble polysaccharides and the development of morphological structures. More studies on their expression patterns and functions in the future could be used to elucidate the molecular mechanisms that regulate polysaccharide synthesis and secondary metabolism in *D. officinale*.

### Cellulose synthase genes and their differential expression patterns in *D. officinale*

Soluble polysaccharides are synthesized from monosaccharides such as mannose, glucose, galactose, arabinose, rhamnose, and others (Zha et al., 2007). Mannose is also the major component of polysaccharides from *Dendrobium* species such as *D*. *officinale, D*. *huoshanense, D*. *nobile, D*. *fimbriatum*, and *D*. *chrysotoxum* (Fan et al., 2009; Luo et al., 2010; Meng et al., 2013; He et al., 2015). Mannans are also promising bioactive polysaccharides for use in drugs (Alonso-Sande et al., 2009). Many studies have proven that CesA superfamily genes are involved in the biosynthesis of mannan polysaccharides (Liepman et al., 2005; Lerouxel et al., 2006).

The CesA superfamily is classified into one cellulose synthase (CesA) family and nine cellulose synthase-like (Csl) families, namely CslA/B/C/D/E/F/G/H/J. Among them, CslF, CslH, and CslJ are specific to monocotyledonous plants while CslB and CslG are found exclusively in dicotyledonous plants (Richmond and Somerville, 2000; Suzuki et al., 2006). Several studies have demonstrated that the Csl families are involved in the biosynthesis of mannan polysaccharides. For example, CslA subfamily members encode β-1,4-mannan synthase (Liepman et al., 2005; Yin et al., 2009), CslC subfamily members encode β-1,4-glucan synthase (Cocuron et al., 2007), while CslF and CslH subfamily members participate in the biosynthesis of β-(1,3;1,4)-D-glucan (Nemeth et al., 2010; Burton et al., 2011; Taketa et al., 2012). The function of the remaining subfamilies members is still unknown. We identified 89 CesA-related genes in the transcriptome database (Additional file 5), which were classified into one CseA family and nine Csl families, including CslB and CslG families. Among these CesA genes, 37 genes showed differential expression between adult plants with juvenile seedlings. These differentially expressed CesA genes only contained five CslA families (Table 5). Furthermore, the up-regulated genes were only found in CslE and CslG families while the down-regulated genes were found exclusively in CslA, CslD, and CslG families. The number of down-regulated genes exceeded that of up-regulated genes. We speculate that these up-regulated CslE and CslG family genes might encode some enzyme responsible for the synthesis of mannan polysaccharides in the stem of *D. officinale*. However, these down-regulated CslA, CslD, and CslG family genes might participate in the synthesis of the backbones of polysaccharides to build plant cell walls and other morphological structures in juvenile seedlings.

### Transcription factors involved in polysaccharide biosynthesis and other secondary metabolism

TFs play diverse roles in regulating the activity of polysaccharide biosynthesis and other secondary metabolism pathways. For example, *Arabidopsis* MYB58 and MYB63, as well as their ortholog PtrMYB28 from *Populus tricocarpa*, are transcriptional activators of the lignin biosynthetic pathway, whereas *Eucalyptus grandis* EgMYB2 and *Pinus taeda* PtMYB4 are involved in the regulation of the entire secondary wall biosynthetic program (Zhong and Ye, 2009). MYB75, which acts as a repressor of the lignin branch of the phenylpropanoid pathway, interacts with another secondary cell wall regulator, the KNOX TF, KNAT7. Together, they form functional complexes to regulate secondary cell wall deposition and to integrate the metabolic flux through the lignin, flavonoid, and polysaccharide pathways in *Arabidopsis* (Bhargava et al., 2010). Overexpression of PAP1, a MYB TF from *Arabidopsis*, resulted in strongly enhanced expression of phenylpropanoid biosynthesis genes as well as enhanced accumulation of lignin, hydroxycinnamic acid esters and flavonoids (Borevitz et al., 2000). In our study, 82 MYB TFs were found to be differential expression in both developmental stages, including 35 up-regulated TFs and 47 down-regulated TFs (Table 6). The up-regulated TFs were probably related with some aspect of secondary metabolism, including polysaccharide and alkaloid biosynthesis, and down-regulated TFs were likely related with morphogenesis, including cell wall formation. In recent years, many WRKY genes have been isolated from medicinal plants and have been shown to play an important role in secondary metabolism. *CjWRKY1* from *Coptis japonica* Makino was the first regulator identified in the biosynthesis of berberine, a benzylisoquinoline alkaloid, and transient expression of *CjWRKY1* in *C. japonica* protoplasts increased the level of transcripts of berberine biosynthetic genes (Kato et al., 2007). *AaWRKY1* from *Artemisia annua* L. could activate Amorpha-4, 11-diene synthase to regulate artemisinin biosynthesis (Ma et al., 2009). *SUSIBA2*, a WRKY TF from *Hordeum vulgare* cv. “Pongo,” was participated in sugar signaling by binding to the sugar-responsive elements of the iso1 promoter (Sun et al., 2003). In our study, 60 WARK TFs were discovered in the DEGs database (Table 6). There have been reports that AP2/ERF family TFs play an important role in plant secondary metabolism. For example, overexpression of the JA-inducible AP2/ERF-domain TF ORCA3 of *Catharanthus roseus*, led to increased expression of several metabolic biosynthetic genes and consequently increased the accumulation of terpenoid indole alkanoids in suspension cells (van der Fits and Memelink, 2000). The NIC2/ORCA3 ERF subfamily from *Nicotiana tabacum* was independently recruited to regulate jasmonate-inducible secondary metabolism in distinct plant lineages (Shoji et al., 2010). In our study, 66 AP2/ERF TFs were discovered in the DEGs database and differentially expressed in the two developmental stages: 21 were up-regulated and 45 were down-regulated (Table 6). In plants, bHLH and bZIP TFs were also isolated and confirmed to regulate secondary metabolism. CrMYC2 belongs to the bHLH TF family and regulates ORCA gene expression, and the AP2/ERF-domain TFs, ORCA2, and ORCA3, in turn regulate a subset of alkaloid biosynthesis genes in *C. roseus* (Zhang et al., 2011). In our work, 75 HLH TFs were discovered. All these TF types known to be involved in regulating secondary metabolism were found in our dataset. They may play important roles in *D. officinale* development, stress responses and secondary metabolism.

## Conclusions

*D. officinale* is a very important Chinese medicinal herb. A total of 145,791 unigenes were obtained in two transcriptome databases of *D. officinale*, 135 of which were involved in fructose and mannose metabolism. In addition, 430 glycosyltransferase and 89 cellulose synthase genes were identified. Comparative analysis of the transcriptome in juvenile seedlings and adult plants revealed a total of 32,794 DEGs that were mainly correlated with metabolic pathways and the biosynthesis of secondary metabolites. A total of 170 glycosyltransferase genes, 37 cellulose synthase genes and 627 transcription factors showed differential expression. This data could be used to investigate pathways associated with polysaccharide biosynthesis and various secondary metabolites in *D. officinale*.

## Author contributions

JZ performed the bioinformatics analyses and drafted the manuscript. CH carried out the experiments. KW and ZY cultured and provided the experimental material. JT critically evaluated the protocol and data, interpreted it, and revised the manuscript. SZ and XZ participated in the qRT-PCR experiment. HX performed the bioinformatics analyses. JD designed the study and revised the manuscript. All authors read and approved the final manuscript.

### Conflict of interest statement

The authors declare that the research was conducted in the absence of any commercial or financial relationships that could be construed as a potential conflict of interest.

## Supplementary material

Additional file 1 **Primer sequences for qRT-PCR**.

Additional file 2 **Metabolic pathway analysis result for unigenes by KEGG annotation**.

Additional file 3 **The lists of carbohydrate-active related unigenes**.

Additional file 4 **The category and number of GTs family in *D. officinale, A. thaliana*, and *O. sativa***.

Additional file 5 **The lists of CesA family related unigenes in *D. officinale***.

Additional file 6 **Specific expression of genes in Dendrobium-1**.

Additional file 7 **Specific expression of genes in Dendrobium-2**.

Additional file 8 **Gene set enrichment analysis in the comparison of Dendrobium-1 vs. Dendrobium-2 by KEGG**.

Additional file 9 **The main biological process for specific unigenes in Dendrobium-1 (A), specific unigenes in Dendrobium-2 (B), up-regulated unigenes (C), down-regulated unigenes (D) in Dendrobium-1 vs. Dendrobium-2**.

Additional file 10 **Lists of DEGs related to glycosyltransferases genes**.

Additional file 11 **Differential expression genes related with GTs in *D. officinale* DEGs. (A)** Heat map analysis of 18 GT-related genes in the comparison of Dendrobium-1 vs. Dendrobium-2. Each row represents a gene. Expression differences are shown in different colors. Red means high expression and green means low expression. **(B–D)** Validation of RNA-sequencing results by real-time PCR. The gene expression in Dendrobium-1 (blue bars), and Dendrobium-2 (red bars). The y-axis indicates relative normalized expression in the Dendrobium-1 and Dendrobium-2 samples. The relative normalized expression of 18 genes in Dendrobium-1 was calibrated as zero.

## Abbreviations

BLAST: Basic local alignment search tool
CBM: Carbohydrate-Binding Module
cDNA: Complementary DNA
CesA: Cellulose synthase
CE: Carbohydrate Esterase
*Csl*: Cellulose synthase-like
COG: Clusters of Orthologous Groups
DEG: Differential expression gene
DGE: Digital Gene Expression
EST: expressed sequence tag
gDNA: Genomic DNA
GH: Glycoside Hydrolase
GO: Gene Ontology
GT: Glycosyltransferase
FDR: False discovery rate
KEGG: Kyoto Encyclopedia of Genes and Genomes
NCBI: National Centre for Biotechnology Information
Nr: Non-redundant protein database
PL: Polysaccharide lyase
qRT-PCR: Quantitative real-time PCR
RPKM: Reads per kilobase of transcript per million reads mapped
SRA: Sequence Read Archive
TF: Transcription factor
TGICL: TGI Clustering Tool
UGT: UDP glycosyltransferase.
