# Supplementary figures and images for: Transcriptome Analysis of Dendrobium officinale and its Application to the Identification of Genes Associated with Polysaccharide Synthesis
Source: Front Plant Sci. 2016 Feb 5;7:5. doi: 10.3389/fpls.2016.00005 (PMC4742539; doi:10.3389/fpls.2016.00005)

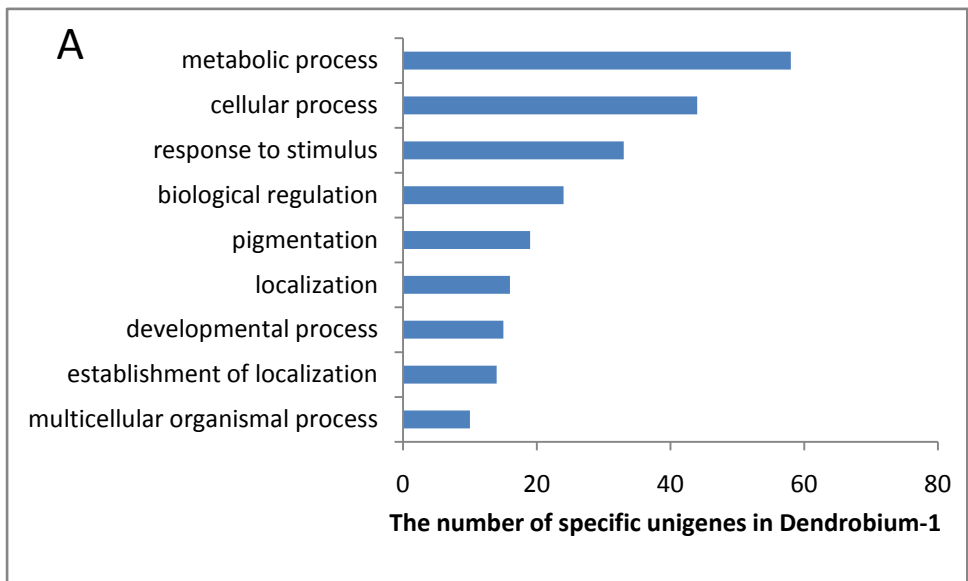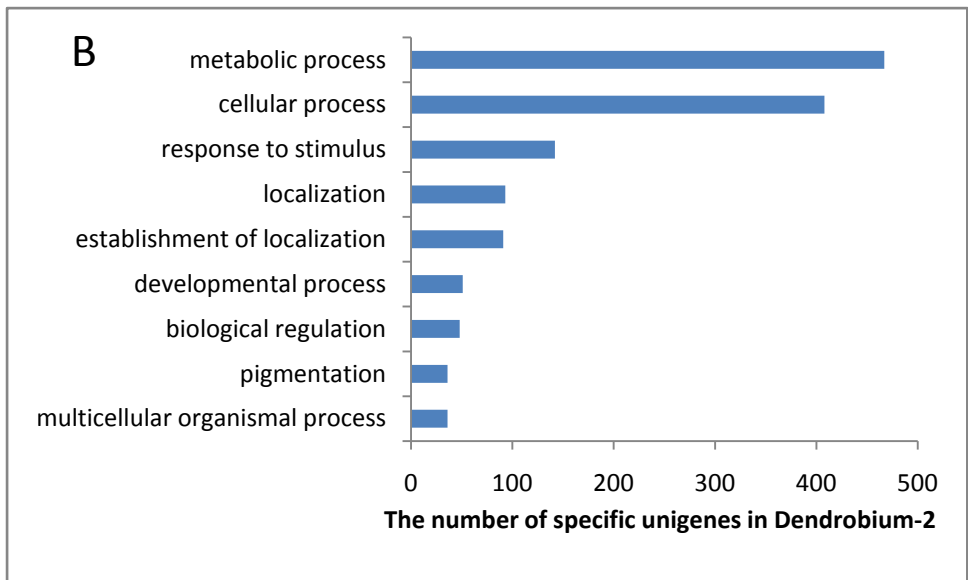

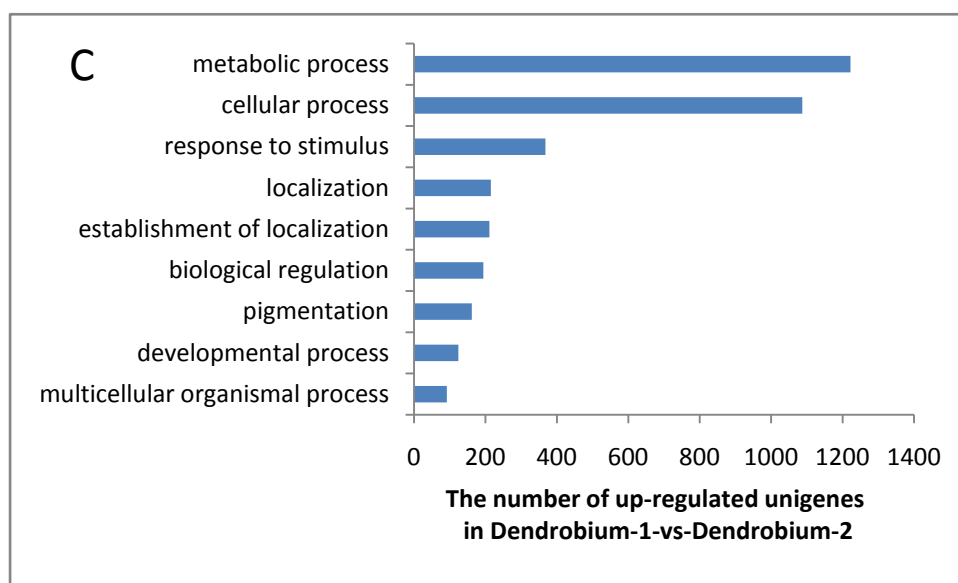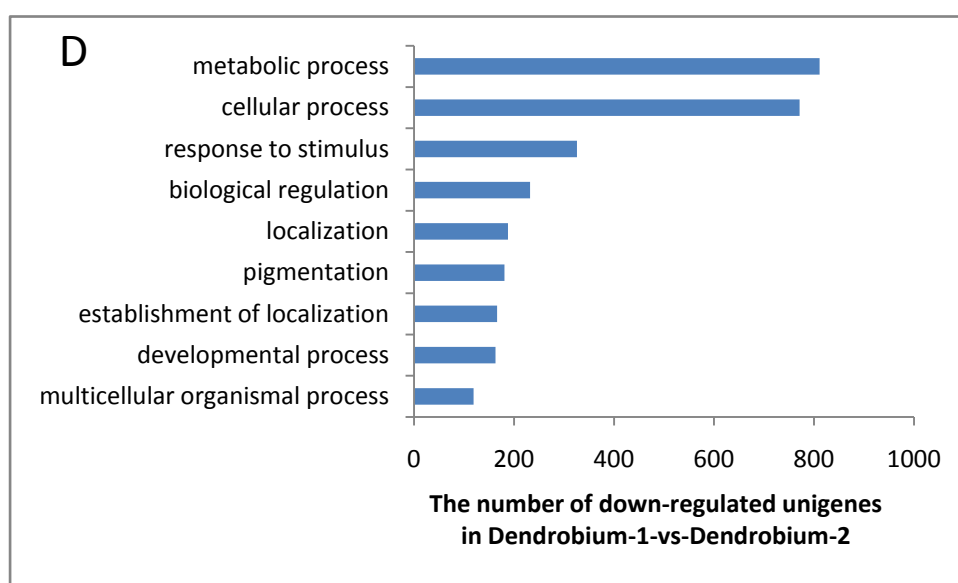

Supplement: Additional file 9 — The main biological process for specific unigenes in Dendrobium-1 (A), specific unigenes in Dendrobium-2 (B), up-regulated unigenes (C), down-regulated unigenes (D) in Dendrobium-1 vs. Dendrobium-2. [file DataSheet9.PDF]

A . The heat map analysis of GTs related genes

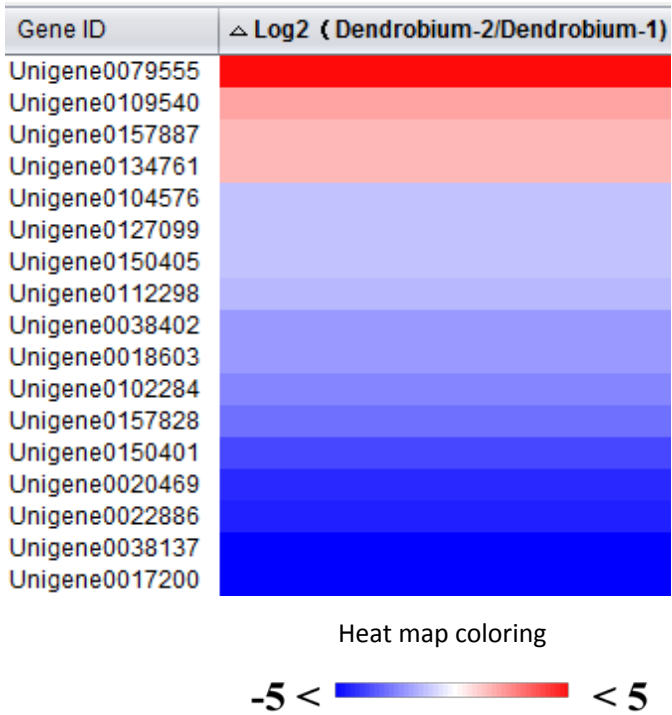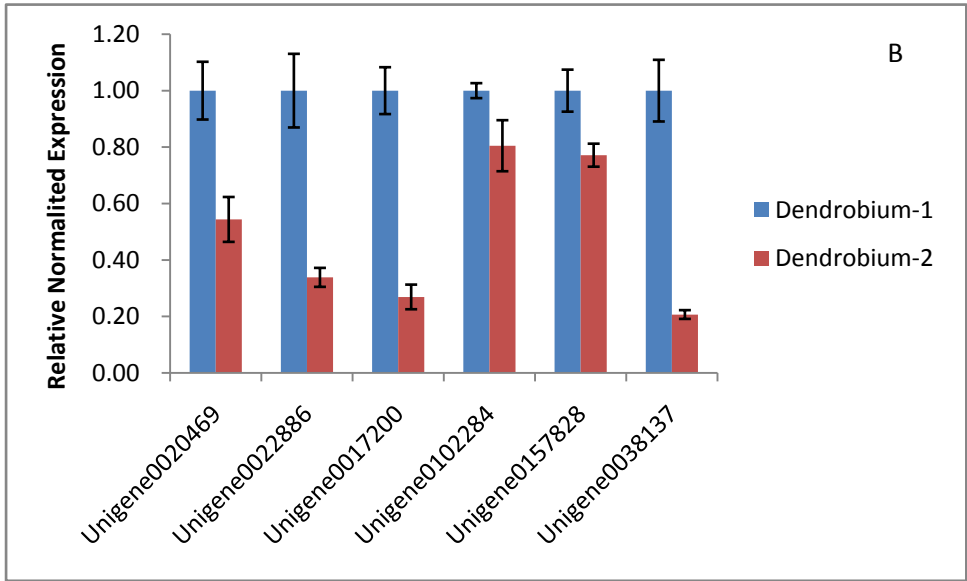

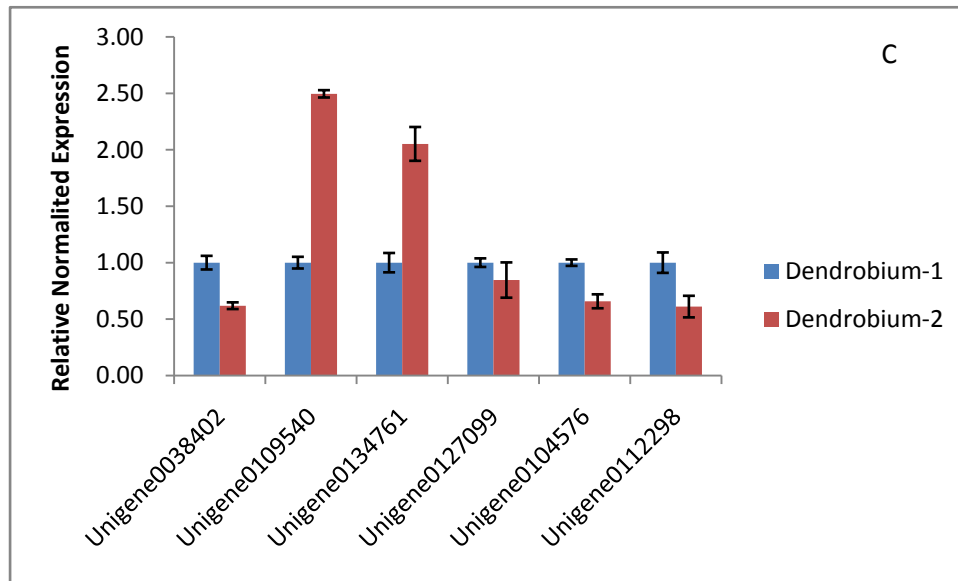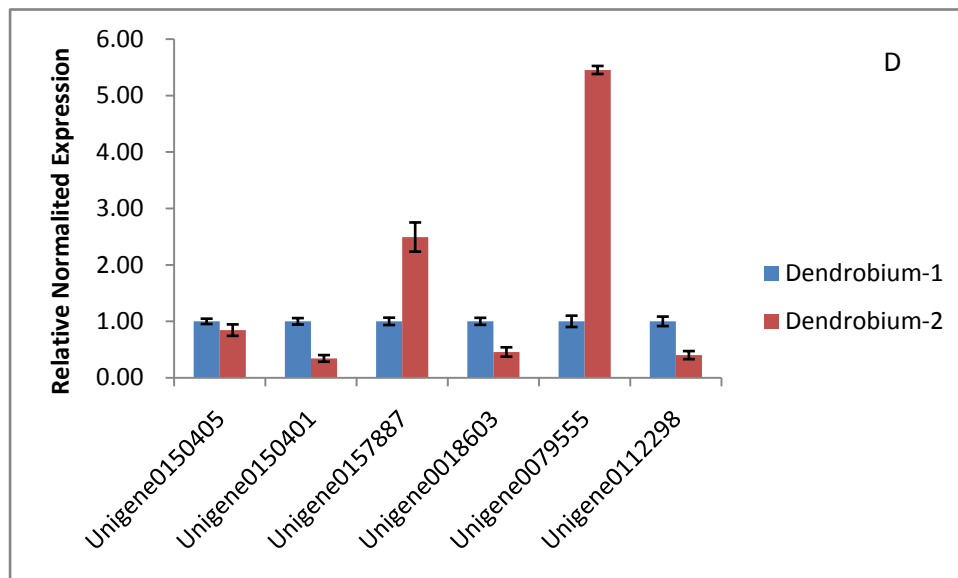

Supplement: Additional file 11 — Differential expression genes related with GTs in D. officinale DEGs. (A) Heat map analysis of 18 GT-related genes in the comparison of Dendrobium-1 vs. Dendrobium-2. Each row represents a gene. Expression differences are shown in different colors. Red means high expression and green means low expression. (B–D) Validation of RNA-sequencing results by real-time PCR. The gene expression in Dendrobium-1 (blue bars), and Dendrobium-2 (red bars). The y-axis indicates relative normalized expression in the Dendrobium-1 and Dendrobium-2 samples. The relative normalized expression of 18 genes in Dendrobium-1 was calibrated as zero. [file DataSheet11.PDF]
